# Affective and Clinical Outcomes Related to Pain After Graded Motor Imagery in Patients With Chronic Shoulder Pain: A Pre–Post-Single-Group Study

**DOI:** 10.1155/rerp/7355866

**Published:** 2024-12-20

**Authors:** Felipe Araya-Quintanilla, Héctor Gutiérrez-Espinoza, Guillermo Méndez-Rebolledo, Iván Cavero-Redondo, Celia Álvarez-Bueno, Dimitrios Stasinopoulos

**Affiliations:** ^1^Escuela de Kinesiología, Facultad de Odontología y Ciencias de la Rehabilitación, Universidad San Sebastián, Santiago, Chile; ^2^One Health Research Group, Universidad de las Americas, Quito, Ecuador; ^3^Escuela de Kinesiología, Facultad de Salud, Universidad Santo Tomás, Talca, Chile; ^4^Universidad de Castilla-La Mancha, Facultad de Enfermeria, Cuenca, Spain; ^5^Facultad de Ciencias de la Salud, Universidad Autónoma de Chile, Talca, Chile; ^6^Department of Physiotherapy, Faculty of Health and Caring Sciences, University of West Attica, Athens, Greece

**Keywords:** catastrophization, chronic pain, graded motor imagery, shoulder pain

## Abstract

**Objective:** The aim of this study was to assess at 6-month and 1-year follow-up the effect of graded motor imagery (GMI) in addition to usual care on the affective and clinical outcomes in patients with chronic shoulder pain.

**Methods:** A pre–post-intervention single-group study was conducted. One hundred forty-eight patients with chronic shoulder pain were included. All participants received a 6-week GMI program in addition to usual care. The primary outcome assessed was pain intensity using visual analog scale (VAS), the secondary outcomes were fear of movement with the Tampa Scale of Kinesiophobia (TSK), catastrophization with the pain catastrophization scale (PCS), shoulder flexion active range of motion (AROM) with a goniometer, and central sensitization with the central sensitization inventory (CSI). All outcomes were assessed at baseline and 6-month and 1-year follow-up.

**Results:** At 6 months, GMI showed to be statistically significant for all outcomes assessed (*p* < 0.001). At 1-year follow-up, the VAS showed a decrease of 3.3 cm (*p* < 0.001), TSK showed a decrease of 16.1 points (*p* < 0.001), PCS showed a decrease of 17.4 points (*p* < 0.001), AROM showed an increase of 29.9° (*p* < 0.001), and CSI showed a decrease of 17.9 (*p* < 0.001).

**Conclusions:** At medium- and long-term follow-up, the individuals who received the GMI program in addition to usual care showed a clinically and statistically significant change for all outcomes assessed. Further studies, including clinical trials, are needed to confirm our findings.

## 1. Introduction

Chronic shoulder pain is one the most common musculoskeletal complaints in the general population and, for the most part, can be managed in primary care [1]. However, in some patients with persistent pain, a clear origin of the nociceptive process is lacking [2]. Some authors have defined it as a complex syndrome that cannot be explained by anatomic injuries or tissue damage [2, 3]. Regarding this, the scientific evidence has shown that the degree of chronic pain is influenced by the beliefs, attitudes, expectations, and socioemotional components related to the central nervous system [4, 5]. In this sense, one of the key factors to explain the chronic pain is the central sensitization (CS). Currently, the rate of pain sensitization is high regardless of the specific etiology, reaching a level of 24% in subjects with shoulder impairment [6]. In these patients, the CS has been proposed to explain the clinical characteristics associated with chronic shoulder pain and is a cause of increased symptoms through an increase in the nociceptive signal from the central nervous system [2, 7].

Currently evidence has showed that patients with chronic pain showed high levels of pain-related affective components such as anxiety, stress, depression, fear of movement, and catastrophizing [8–10]. These findings are related to a high level of neuronal excitability in the somatosensory cortex, insula, and amygdala. Impairments in these brain areas lead to fear processing, increased pain catastrophizing, negative patient behavior, and pain processing dysfunction, which determines the persistence of symptoms in patients with chronic musculoskeletal pain [11–13].

Some studies have analyzed the effect of graded motor imagery (GMI) in the short term on chronic musculoskeletal pain [14–16]. Additionally, recent systematic reviews have shown that the visual observation of movement and mirror therapy are effective therapeutic interventions in some populations with chronic musculoskeletal pain [16–18]. One study concluded that motor imagery may be effective for pain relief and improve range of motion; nevertheless, the conclusion is based on a limited certainty of evidence and has not been studied in all chronic musculoskeletal pain populations [18]. According with this, the evidence about GMI effects in patients with chronic shoulder pain is scarce [19–21]. Previous studies have shown favorable results for mirror therapy and GMI on clinical outcomes; however, there are no studies with a follow-up after the intervention and long-term effects are unknown in these patients.

To our knowledge, no previous studies have shown the clinical effects of a GMI program at medium- or long-term follow-up in patients with chronic shoulder pain. Thus, the aim of this study was to assess at six-month and 1-year follow-up the effect of GMI program in addition to usual care on the affective and clinical outcomes of pain in patients with chronic shoulder pain syndrome.

## 2. Methods

### 2.1. Study Design and Patients

This pre–post-intervention single-group study was reported based on the Strengthening the Reporting of Observational Studies in Epidemiology (STROBE) statement [22]. This study was approved by the Ethics Committee of the University of the Americas (approved Oct 2018). Between October 2018 and April 2022, 148 patients with chronic shoulder pain syndrome were prospectively recruited and written informed consent was obtained for all patients. They were recruited from the Physiotherapy Department of a private clinic in Santiago, Chile. The diagnosis was performed by an orthopedic surgeon based on imaging studies that included anteroposterior projection radiographs, axial and outlet, ultrasonography of soft tissue, and magnetic resonance imaging. The inclusion criteria were (i) patients older than 60 years with shoulder pain for a duration of at least 6 months secondary to tendinopathy and/or partial rotator cuff tear, (ii) patients able to follow simple orders, and (iii) patients able to sign the informed consent form. Conversely, patients with other pathologies of the shoulder joint complex (full thickness rotator cuff, adhesive capsulitis, and glenohumeral instability); history of acute trauma, previous surgery, or previous fracture in the affected shoulder; history of radiotherapy on the same side as the affected shoulder; rheumatoid arthritis or any other inflammatory disorder of the joints; or symptomatic cervical spine pathology were excluded.

### 2.2. Usual Care

All patients were prescribed to usual care, using nonsteroidal anti-inflammatory drugs (celecoxib 200 mg, twice a day, for 14 days) and standard medical education, and subsequently were referred for physiotherapy treatment, as part of the protocol of the study.

### 2.3. GMI Program

All patients received a GMI program based on the manual proposed by Moseley et al. [23], in addition to usual care. The entire GMI program was performed three times a week during six consecutive weeks and includes the following three steps: left–right limb judgments (implicit motor imagery), imagined limb movements (explicit motor imagery), and mirror therapy. All complete steps and procedures were detailed in our previous study [20]. For the first step, the patients train by looking at left and right images of different body parts in different positions. The Recognise application created by the Neuro Orthopedic Institute (NOI) in a tablet was used. For the second step, the patients train by “imagining movements in the direction of shoulder flexion, abduction, and rotation” without actively performing them. In this way, we activate the premotor cortex without generating movement and causing pain in the patients. Finally, for the last stage, the patients train with mirror therapy; mirror therapy involves using a mirror to observe the movement of the unaffected part of the body. This creates the illusion that the shoulder moves without pain. The patients performed mirror therapy once per session for 30 min. Finally, patients did not receive instruction on how to continue the therapy at home.

### 2.4. Outcome Measures

For this study, three evaluations were performed and considered for the analysis, at the beginning, 6-month, and 1-year follow-up. Assessment consisted of a physical examination of the affective components of pain, shoulder flexion range of motion, and CS level. All measurements were performed by two external physiotherapists, and both assessed the same number of patients. For the evaluations, patients were called via telephone or email by a physiotherapist part of the research team to coordinate the evaluations.

### 2.5. Primary Outcome

Pain intensity was assessed using the visual analog scale (VAS), which consists of a horizontal line 10 cm in length, where the left end represents 0 or “painless” and the right end 10 or “worst pain imaginable.” The patient was asked to mark with a vertical line the magnitude of the pain felt at the time of the evaluation. This is a one-dimensional, simple, and reproducible assessment method [24]. The minimum clinically important difference (MCID) has been reported as 1.1 cm for shoulder pain [25].

### 2.6. Secondary Outcomes

#### 2.6.1. Kinesiophobia

For kinesiophobia or pain-related fear of movement, the original 17-item Tampa Scale of Kinesiophobia (TSK) was used [26]. Each item is scored on a 4-point Likert-type scale that ranges from *strongly agree* (1) to *strongly disagree* (4). Total scores range from 17 to 68, and higher scores indicate more fear of movement and/or (re) injury. In patients with shoulder pain, the minimal detectable change for the TSK is reported to be 5.6 points [26].

#### 2.6.2. Pain Catastrophization

Pain catastrophization was assessed with the pain catastrophization scale (PCS). The PCS is a self-administered questionnaire that evaluates inappropriate coping strategies and catastrophic thinking about pain [27]. The PCS uses a Likert scale of 13 items, comprising three dimensions: (a) rumination, (b) magnification, and (c) hopelessness. This scale could range between 13 and 62 points, with low scores indicating low catastrophization. In patients with shoulder pain, the minimal detectable change for the PCS is 9.1 points [28].

#### 2.6.3. Shoulder Active Range of Motion (AROM)

Shoulder flexion AROM was measured using a universal goniometer (Plastic BASELINE Model 12–1000, White Plains, New York, United States), with a precision of ±2°. The movement was measured three times, and the mean of these measurements was used for the analysis. The goniometer has good intrarater reliability when consistent body landmarks are used (intraclass correlation coefficient ≥ 0.85) [29]. The minimal detectable change for shoulder flexion has been reported as 8° [29].

#### 2.6.4. CS

CS was assessed with the central sensitization inventory (CSI) [30]. The questionnaire includes 25 items, and scores were assigned from 0 (*best*) to 4 (*worst*) for each item. The maximum total score was 100 points, and a higher score indicates more CS. Values > 40 points indicate moderate CS [30]. This questionnaire has psychometric strength, clinical utility, and reproducibility [31]. The MCID for CSI has not been reported.

### 2.7. Statistical Analysis

The parametric distribution of the continuous variables was checked using both the Kolmogorov–Smirnov test and graphical procedures (normal probability Q-Q plot). Data were presented as mean and standard deviation for continuous variables and as number and percentage for categorical variables. Univariate tests for intragroup comparisons were conducted to analyze differences between pre- and posttests (Δ). Additionally, we calculated partial eta squared (*ƞ*^2^) for the effect size of the postoperative program, considering the effect as small (0.0–0.13), medium (0.13–0.26), or large (> 0.26) [32]. Analysis of variance (ANOVA) was used to determine the differences between the baseline data and at 6-month and 1-year follow-up. Previously, a homoscedasticity analysis was used with the Levene test to assess the homogeneity of variances. To identify differences between the three measurements, the post hoc power correction of the Bonferroni test was used. The significance level was set at *p* < 0.05. Finally, data were analyzed using the Statistical Package for the Social Sciences (SPSS) Version 26 (SPSS Inc., Chicago, Illinois, United States).

## 3. Results

The baseline information characteristics of the sample are shown in Table 1. Eighty-one patients are women (54.8%), and the baseline CSI is 48.3 points, which indicates a higher level of CS. The mean duration of symptoms was 61.2 months. The most common type of shoulder condition was partial rotator cuff tear (57.5%). Additionally, there was no loss or withdrawal during the study. At the end of the study, no patient informed the researchers of any complications associated with the treatment received.

Table 2 shows baseline and 6-month and 1-year follow-up outcomes. At 6 months of follow-up, the VAS showed a decrease of 3.2 cm (confidence interval (CI) 95% 2.8–3.5; *p* < 0.001), TSK showed a decrease 15.8 points (CI 95% 12.5–15.9; *p* < 0.001), PCS showed a decrease 29.8 points (CI 95% 12.7–17.2; *p* < 0.001), AROM showed an increase of 24.2° (CI 95% 20.4–31.8; *p* < 0.001), and CSI showed a decrease 12.7 (CI 95% 6.6–13.4; *p* < 0.001).

**Figure 1:**
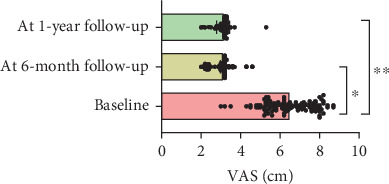
Changes of results between the baseline and 6-month and 1-year follow-up for VAS.

**Figure 2:**
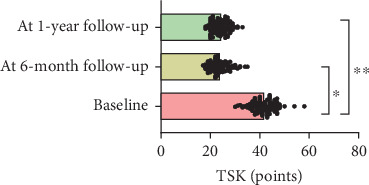
Changes of results between the baseline and 6-month and 1-year follow-up for TSK.

**Figure 3:**
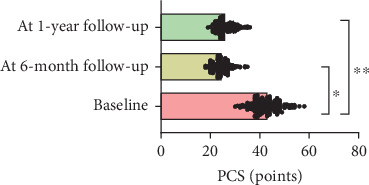
Changes of results between the baseline and 6-month and 1-year follow-up for PCS.

**Figure 4:**
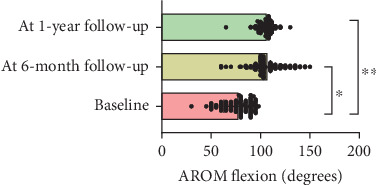
Changes of results between the baseline and 6-month and 1-year follow-up for shoulder AROM.

**Figure 5:**
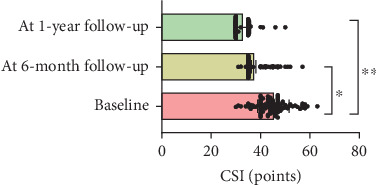
Changes of results between the baseline and 6-month and 1-year follow-up for shoulder CSI.

At 1-year follow-up, the VAS showed a decrease of 3.3 cm (*ƞ*^2^ = 0.7; CI 95% 0.68–0.75; *p* < 0.001), TSK showed a decrease of 16.1 points (*ƞ*^2^ = 0.7; CI 95% 0.76–0.81; *p* < 0.001), PCS showed a decrease of 17.4 points (*ƞ*^2^ = 0.8; CI 95% 0.78–0.83; *p* < 0.001), AROM showed an increase of 29.9° (*ƞ*^2^ = 0.3; CI 95% 0.32–0.45; *p* < 0.001), and CSI showed a decrease of 17.9 (*ƞ*^2^ = 0.3; CI 95% 0.32–0.41; *p* < 0.001) (Figures 1, 2, 3, 4, and 5). At 1-year follow-up, in all outcomes assessed, the values of *η*^2^ showed a large effect size (*η*^2^ > 0.26).

## 4. Discussion

This study is aimed at describing the effect of GMI in addition to usual care on affective and clinical outcomes of pain in subjects with chronic shoulder pain syndrome. The main findings were, at 6-month and 1-year follow-up, the patients who received the GMI program added to usual care showed a clinically and statistically significant change for all outcomes. For most outcome assessed, they met established MCID thresholds.

A recent systematic review showed that a significant proportion of the population across the world experienced shoulder pain daily, yearly, and throughout a lifetime [33]. Nevertheless, there is no reference standard treatment for patients with chronic shoulder pain [34]. Additionally, the prognosis varies widely between individuals, but on average, 50% of people with shoulder pain still report symptoms 6 months after presenting in primary care [35]. For these reasons, some evidence suggests that the management for chronic musculoskeletal shoulder pain should consider other contemporary strategies and should be multidisciplinary [36, 37]. However, there is still uncertainty about how to apply exercise prescription or the best treatment strategies for these patients [38]. This could be explained because the change in pain signaling pathways leading to an increased responsiveness of nociceptive neurons to their normal input referred as pain sensitization. Therefore, patients may not respond to traditional exercise in an early phase or stage of treatment [38–41]. A recent meta-analysis showed that pain sensitization has a high rate in patients with musculoskeletal chronic shoulder pain, and this may lead to worse clinical outcomes after first-line treatment [6].

The positive effects reported in our study are in accordance with previous studies in patients with chronic shoulder pain [20, 42]. One study showed that a short-term GMI program improves the affective components of pain and shoulder flexion AROM in patients with chronic shoulder pain syndrome [20]. Another study showed the immediate effect of mirror therapy improves pain, pain catastrophization, fear avoidance, and shoulder flexion AROM in patients with shoulder pain [42]. Both studies only showed short-term effects. To our knowledge, no previous studies have showed in the medium- or long-term follow-up the clinical effects of a GMI program in these patients.

Our results can be explained based on the mechanisms of GMI, which could be attributed to activating the cortical networks in different areas [43]. One of the main effects attributed to GMI is that it desensitizes the hypersensitive nervous system and thus helps in reducing fear of movement [42]. For instance, some studies showed a decrease on neural activity in somatosensory cortex, amygdala, and insular cortex, areas related to the affective processing of pain [14, 42]. In this sense, the GMI is an intervention that can help reduce the central nervous system's sensation of threat or damage [23, 43]. Finally, these immediate changes may allow a quicker transition to apply other therapeutic interventions, such as exercises or manual therapy techniques.

Previous studies and systematic reviews have analyzed the effectiveness of GMI in other musculoskeletal conditions [15, 18, 44–46]. One study showed that GMI is more effective for pain relief than usual physiotherapy in patients with chronic knee pain [15]. Another systematic review showed that motor imagery is more effective for pain relief and improving range of motion than standard rehabilitation in chronic musculoskeletal pain conditions [45].

Additionally, a recent study showed that addition of GMI to physiotherapy treatment increases the abduction and external rotation of the shoulder and decreases the pain intensity in patients with frozen shoulder [44]. However, only two studies described the medium-term effects of GMI in different musculoskeletal chronic pains [47, 48]. In this sense, the lack of follow-up of this intervention in patients with chronic shoulder pain leaves doubts about its long-term effects.

The clinical applicability of the GMI program in addition to usual care is the main strength of this study. Our results could be a guide to physiotherapist to perform a safe therapeutic intervention for patients with chronic shoulder pain. However, our study has some limitations. First, the absence of a control group and the lack of control for confounding factors make it difficult to establish a cause–effect of GMI program on the outcomes analyzed. Second, blinding of the physiotherapists and participants was not possible due to the nature of the studied interventions. Third, self-report questionnaires were used for assessment, which are prone to subjectivity and recollection bias. These limitations should be considered when attempting to extrapolate our findings to the treatment of all patients with chronic shoulder pain.

## 5. Conclusions

At medium- and long-term follow-up, the patients with chronic shoulder pain who received the GMI program in addition to usual care showed a clinically and statistically significant change for all outcomes assessed. Based on our findings, we recommended that physiotherapists incorporate GMI for clinical management of these patients. Finally, further studies, including clinical trials, are needed to confirm our findings.

## Figures and Tables

**Table 1:** Baseline characteristics of patients with chronic shoulder pain.

**Characteristics**	**Patients (** **n** = 148**)**
Female, number (%)	81 (54.8)
Male, number (%)	67 (45.2)
Age (years), mean (SD)	68.8 (4.3)
CSI	48.3 (7.4)
Duration of symptoms (months), mean (SD)	61.2 (3.8)
Rotator cuff tendinopathy, number (%)	63 (42.5)
Rotator cuff tear (partial), number (%)	85 (57.5)
Height (m), mean (SD)	1.68 (0.9)
Weight (kg), mean (SD)	72.4 (4.3)
BMI (kg/m^2^), mean (SD)	27.4 (2.1)
Education level, number (%)	
Primary education not complete	27 (18.3)
Primary education	24 (16.2)
Middle education	59 (39.9)
Higher education	38 (25.6)

Abbreviations: BMI: body mass index; CSI: central sensitization inventory; SD: standard deviation.

**Table 2:** Changes of results between the baseline and 6-month and 1-year follow-up.

**Outcome measures**	**Baseline, mean (SE)**	**At 6-month follow-up mean (SE)**	Δ** mean difference (CI 95%)**	**p** ** value**	**At 1-year follow-up mean (SE)**	Δ** mean difference (CI 95%)**	**ƞ** ^2^ ** (CI 95%)**	**p** ** value**
Pain intensity (VAS)	6.5 (0.3)	3.2 (0.8)	3.2 (2.8–3.5)	< 0.001^a^	3.0 (0.8)	3.3 (3.0–3.7)	0.7 (0.68–0.75)	< 0.001^b^
TSK	43.5 (0.4)	27.7 (0.2)	15.8 (12.5–15.9)	< 0.001^a^	27.4 (0.2)	16.1(12.8–17.4)	0.7 (0.76–0.81)	< 0.001^b^
Catastrophization PCS	46.8 (0.5)	29.8 (0.2)	17 (12.7–17.2)	< 0.001^a^	29.4 (0.3)	17.4 (13.1–18.6)	0.8 (0.78–0.83)	< 0.001^b^
Shoulder flexion AROM	79.1 (1.3)	103.3 (1.2)	24.2 (20.4–31.8)	< 0.001^a^	108.1 (1.6)	29.9 (25.2–36.6)	0.3 (0.32–0.45)	< 0.001^b^
CSI	48.3 (0.6)	35.6 (1.0)	12.7 (6.6–13.4)	< 0.001^a^	30.4 (0.6)	17.9 (11.8–18.5)	0.3 (0.32–0.41)	< 0.001^b^

Abbreviations: AROM: active range of motion; CI 95%: confidence intervals 95%; CSI: central sensitization inventory; PCS: pain catastrophizing scale; SE: standard error; TSK: Tampa Scale of Kinesiophobia; VAS: visual analog scale.

^a^Difference between baseline and 6-month follow-up.

^b^Difference between baseline and 1-year follow-up with ANOVA (Bonferroni post hoc correction).

## Data Availability

The datasets used and/or analyzed during the current study are available from the corresponding author on reasonable request.
